# Microbiologic features of prosthetic joint infections at a tertiary referral orthopaedic unit

**DOI:** 10.1007/s11845-025-03933-4

**Published:** 2025-03-13

**Authors:** Stephen Christopher Murphy, Shane P. Russell, James A. Harty, Padhraig O’Loughlin

**Affiliations:** 1 Cork University Hospital, Cork, Ireland; 2 Royal College of Surgeons in Ireland, Dublin, Ireland; 3 University College Cork, Cork, Ireland

**Keywords:** Antibiotics in prosthetic joint infection, Local microbiome, Orthopaedic infection, Prosthetic joint infection

## Abstract

**Background:**

Prosthetic joint infections (PJI) are a potential devasting consequence of arthroplasty surgery. Knowledge of the causative organism and antimicrobial sensitivity increases eradication success rates for PJI.

**Aims:**

This study aimed to: 1) Observe the PJI microbiome at a tertiary referral arthroplasty unit; 2) Make comparison to similar published observations; and; 3) Establish empiric local antibiotic PJI guidelines.

**Methods:**

All patients with positive tissue cultures for PJIs over a 4 year period were included. An electronic microbiology laboratory database search was performed to identify isolated microorganisms, sensitivities and resistances. Time from index procedure to PJI onset was recorded. The identified PJI microbiome was compared to current literature.

**Results:**

86 patients involving 88 joints were included. 56% (*n* = 49) related to hip, 42% (*n* = 37) to knee and 2% (2) to shoulder arthroplasty procedures. Coagulase Negative *Staphylococci* (CoNS) were isolated in 32% of cases, *Staphylococcus aureus* (SA) in 23%, *Enterococcus* species and *Streptococcus* species in 9.0%. 19% of case occurred within 3 months of index surgery, 17% from 3–12 months and 64% after 12 months. The microbiome identified varied comparable studies.

**Conclusion:**

This study describes a local PJI microbiome with contrasting results from comparable studies. Empiric antibiotic guidelines have been established to target treatment and a local PJI register has since been established to improve patient outcomes and antimicrobial stewardship in an era of antibiotic resistance.

## Introduction

Prosthetic Joint Infections (PJI) are a potentially devastating complication of hip and knee arthroplasty associated with life-changing morbidity and mortality [1–4]. The diagnosis and treatment of PJIs are often extremely challenging for patients, physicians and surgeons. Complex surgical and medical eradication measures are often necessary [5–7]. An enormous healthcare cost is also associated with PJIs, which are estimated to consume half of all costs associated with revision arthroplasty surgery [13, 14]. In addition, several studies demonstrate how PJI diagnoses may have devastating emotional impacts on both patients and healthcare providers [8–11].

Intra-operative tissue sampling and laboratory specimen analysis provides essential pathogen data to guide antibiotic therapy and improve eradication success rates. [18] Antibiotic selection, more so than duration, is critical for achieving successful outcomes [19].

A myriad of causative microorganisms have been associated with PJIs [12]. A comprehensive understanding of these causative organisms aids individual healthcare institutions in formulating accurate empirical guidelines for the initial treatment of PJIs. Knowledge of the causative organism is therefore invaluable for the treating multidisciplinary team so that surgical and medical therapies may be tailored to achieve the most successful outcomes [13].

This study aimed to: 1) Observe the PJI microbiome at a tertiary referral arthroplasty unit, making comparison to similar published observations; 2) Informed by this observation, formulate local antimicrobial guidelines for PJIs; and 3) Establish a local PJI register for prospective microbiome observations.

## Methods

A retrospective cohort study design was employed at a tertiary referral arthroplasty institution. A search was performed of the institution’s electronic patient records covering a 4-year period from October 2019. Using the search terms: *“PJI”, “Prosthetic joint infection”, “first stage”, “Second stage”, “1st stage”, “2nd stage”, “Revision”, “Debridement Antibiotics Implant Retention”* and *“DAIR*”, 208 patients were initially identified. Procedures that did not relate to: percutaneous aspirations of septic prosthetic joints, open or arthroscopic washouts of septic prosthetic joints, Debridement Antibiotic and Implant Retention (DAIR) procedures, or revision total knee arthroplasty (TKA) and total hip arthroplasty (THA) procedures were subsequently excluded for data analysis (*n* = 117).

After cross-reference of these procedures with the institution’s electronic laboratory database to include only procedures where specimens yielded positive culture results, 88 diagnosed PJIs were included. These related to 86 patients, as 2 patients had multiple PJIs. A total of 136 procedures were included, relating to these 88 PJI diagnoses (86 patients) as most diagnoses required multiple surgical procedures.

Time from index arthroplasty to first return to theatre for PJI (suspected or diagnosed) was also calculated using electronic health records.

*Microsoft Excel* (Redmond, WA, US) was used for data collection and descriptive statistical analysis.

## Results

### Patients and procedures

**Fig. 1:**
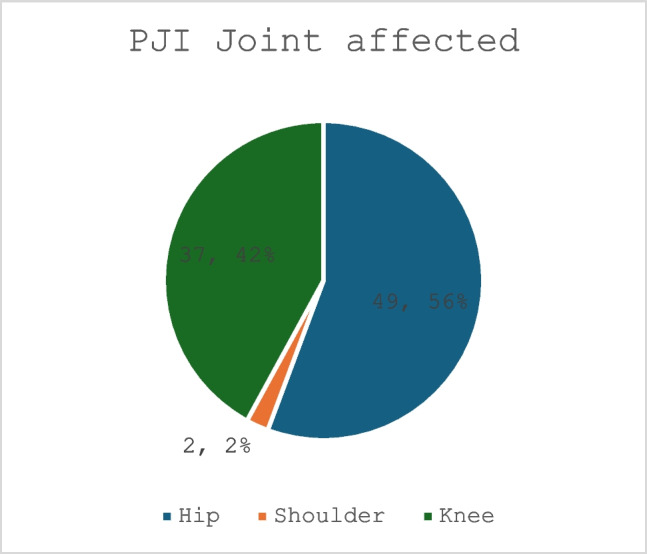
PJI Joint affected

A mean of 2.2 procedures per PJI diagnosis (*n* = 88) were performed. Of these 191 procedures initially identified, 29% (*n* = 55) resulted in no organismal growth and were therefore excluded. The remaining 71% (*n* = 136) of procedures resulted in positive microscopy or positive culture results for one or more microbe. The mean patient age was 71 years (range: 51 to 91 years). 42 PJIs (47%) related to THAs, 4 (5%) related to hip hemiarthroplasties, and 3 (3%) had hip resurfacing prostheses. 37 PJIs (42%) related to TKAs, with the remaining 2 (2%) procedures relating to shoulder arthroplasty diagnoses (Fig. 1).

**Fig. 2:**
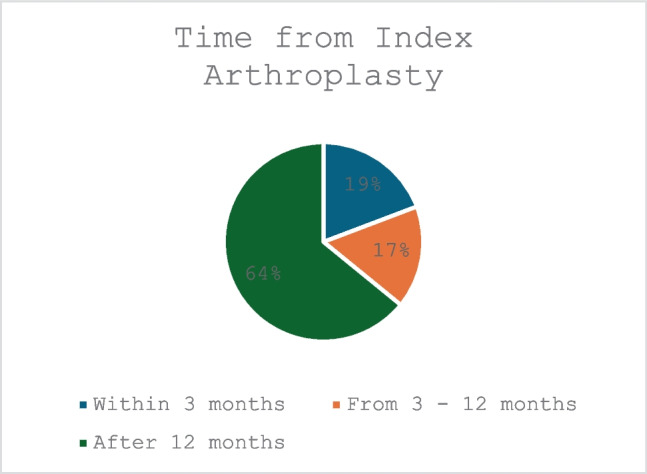
Time from index arthroplasty procedure

Of the 88 patients included, the time elapsed from index arthroplasty procedure to PJI diagnosis was available for 78 (89%) patients. 19% of these occurred within 3 months of index surgery, 17% from 3 to 12 months and 64% after 12 months (Fig. 2).

### Microbiome

**Fig. 3:**
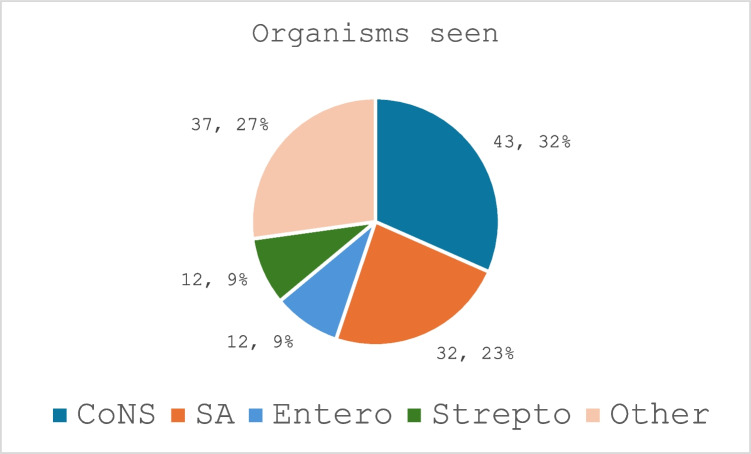
Distribution of Organisms Type Within Microbiome

71% (*n* = 136) of procedures resulted in at least one positive microbial specimen growth. From the overall 191 specimen cohort, 136 s procedures grew one or more organisms whilst there was no organism growth for 55 procedures (29%). A total of 52 distinct organisms were identified. Coagulase Negative *Staphylococci* (CoNS) were isolated in 32% (*n* = 43) of procedures, *Staphylococcus aureus* (SA) in 23% (*n* = 32) and both *Enterococcus* species (Ent.Sp) and *Streptococcus* species (Str.Sp) in 9% (*n* = 12). 27% (*n* = 37) of procedures grew microorganisms grouped for this study as ‘other’. The 5 most common organisms seen in this ‘other’ group were *Proteus mirabilis, Escherichia coli, Pseudomonas aeruginosa, Klebsiella pneumoniae* and *Clostridium perfringens* (Fig. 3).

### Coagulase negative staphylococci

The *Coagulase Negative Staphylococci* (CoNS) group, composed of a variety of low virulence species of the bacterium staphylococcus, accounted for 32% (*n* = 43) of cases. 67.4% (*n* = 29) were seen in the setting of polymicrobial PJIs (pPJI) and 32.6% (*n* = 14) in the setting of monomicrobial PJIs (mPJI).

*Staphylococcus epidermidis* and *Staphylococcus capitis* were the two most commonly isolated species, comprising 38% (*n* = 16) and 23% (*n* = 10) of isolates respectively.

### Staphylococcus aureus

Staphylococcus aureus (SA) was identified in 23% (*n* = 32) of PJIs. mPJI with SA was observed in 59.4% of cases compared to 32.6% of CoNS cases that exhibited a mPJI growth. Methicillin Resistant Staphylococcus Aureus was cultured in 2.3% (*n* = 2) of cases, accounting for 6% of all SA results.

### Enterococcus species and streptococcus species

The third and fourth most commonly identified bacterial group was *Enterococcus* species (Ent.Sp) and *Streptococcus* species (St.Sp), both seen in 9% (*n* = 12) of cases examined. Both groups had equal rates of pPJI and mPJI at 75% (*n* = 9) and 25% (*n* = 3) respectively. Vancomycin-Resistant Enterococcus was seen in 4.5% (*n* = 4) of PJIs, accounting for 33% of Enterococcus infections.

### Other

This group was comprised of 37 different organisms and included fungi such as *candida* species (*n* = 3). Overall, organisms within this group were identified in 27% (*n* = 37) of procedures. An Extended-Spectrum Beta-Lactamase producing bacteria was identified in a single PJI sample (1.1%).

## Discussion

The aims of this study were to: 1) Observe and compare the PJI microbiome at a high-volume revision arthroplasty unit; 2) Utilise those microbiological observations to recommend empiric antibiotics; and 3) Transition from retrospective to prospective PJI microbiome observations at that unit through the establishment of a PJI register.

This study observed a 32% prevalence of CoNS amongst positive PJI cultures, whilst SA accounted for 23%. Similar prevalences of these most common PJI pathogens were observed by Tande et al. (within an aggregate cohort of more than 2,400 Unites States (US) patients with PJIs, identifying a 27% prevalence for both CoNS and SA [2]. Within that study, an 8% prevalence of *Streptococcus* was also observed, similar to the present study’s observation at 9% [2]. The present study, however, observed a higher prevalence of E*nterococcus* species at 9% compared to Tande et al.’s observation of just 3% within the same large cohort [2].

Microbiomes differ across time, populations and geographic regions. [14] Whilst Tande et al. describe geographically distant observations to the present study, Kutubi et al. observed a PJI microbiome amongst a 41 patient cohort between 2015 and 2020 also within the Irish healthcare setting. Similar to the present study, a 36.6% CoNS was observed with a higher SA prevalence at 39%. A lower *Enterococcus* prevalence was observed at 4.8% [15].

Aggarwal et al. compared German and US PJI microbiomes. They found a higher prevalence of CoNS (39.3% versus 20.2%) in the German cohort and a lower prevalence of SA (13% versus 31.0%). The German cohort demonstrated a more favourable antibiotic resistance profile. These results were mirrored by large British and Swedish cohort observations, again demonstrating a high prevalence of CoNS PJI [16–18] (Table 1).

**Table 1:** Literature comparison of monomicrobial PJIs

	Present Study (Ireland)	Kutubi (Ireland)	Aggarwal (Germany)	Aggarwal (US)	Tande (US)
CoNS	15.9%	36.6%	31%	13%	27%
SA	21.6%	39%	20.2%	39.3%	27%
Enterococcus	3.4%	4.8%	3.9%	7%	3%
Streptococcus	3.4%	2.4%	5.8%	6.5%	8%
Polymicrobial PJIs	42%	n/a	7.4%	3.5%	15%

Subsequent to the microbiome features set out by this study, in collaboration with microbiology and infectious disease (ID) team colleagues, well-informed empiric antimicrobial guidelines have now been implemented at this study’s location. Local empiric antibiotic guidelines now recommend initial treatment with intravenous ceftriaxone and vancomycin for suspected PJIs, prior to pathogen identification. This study did not encounter any PJI caused by microorganisms resistant to this dual antibiotic regime.

Empowered by the findings of this study, a prospective register has now been established at this study’s institution. Further efforts are underway to engage with a national and/or international register. In addition, coincident with the investigations and discussions of this study and others, a new multidisciplinary team has been formed at this unit for prospective PJI case management.

Whilst not a primary aim of this study, it was noted that a higher proportion of patients (56%) commenced PJI treatment, at more than 12 months after index arthroplasty. This was in contrast to Philips et al. who studied over 10,000 patients, finding just 36% of PJIs arose after twleve months. Time from primary arthroplasty to PJI diagnosis has been correlated with both eradication success rates and causative pathogens and further studies may evaluate potential causes and consequences for this observation. [19, 20].

This study had several limitations. Firstly, a potentially incomplete patient search method was employed. Whilst a thorough search of the institution’s inpatient and theatre records was performed, some patients who had procedures such as joint aspirations carried out in the Emergency Department or on inpatient wards may have been omitted. However, the aim of this study was to describe the microbiome of PJI diagnoses from positive microbial cultures. Whilst omitting such cases may influence the specimen positivity rate, this rate was outside the scope of this study.

Secondly, the authors identified a 3-month block of missing electronic patient data within the observation period. Best efforts to recover this data were unsuccessful, however, it is felt unlikely this period would contain data that would significantly influence findings given the 4-year observation period. In addition, due to unavailability of patient data from referring private institutions, time from index arthroplasty to PJI surgery was not calculable in 11% (*n* = 10) of cases.

This study represents a relatively large observation of positive specimens in the setting of PJI given the low rate of infection associated with arthroplasty surgery [1]. Whilst Philips et al. examined a cohort of more than 10,000 hip and knee arthroplasty patients, just 75 PJIs (< 0.75%) were included for analysis. The present study was performed at a unit providing both tertiary elective arthroplasty surgery and Level 1 Trauma Centre care for a population of 1,100,000, thereby capturing a large regional PJI microbiome.

With the rapidly increasing incidence of PJIs within our populations, in an era of advancing antimicrobial resistance, healthcare providers may be able to implement more effective and bespoke treatment strategies utilising a prospectively updated and accurate regional microbiome profile.
